# Health technology assessment of tests for SARS-CoV-2 and treatments for COVID-19: A proposed approach and best-practice recommendations

**DOI:** 10.1017/S0266462323000223

**Published:** 2023-04-24

**Authors:** Jamie Elvidge, Ashley Summerfield, Saskia Knies, Bertalan Németh, Zoltán Kaló, Wim Goettsch, Dalia M. Dawoud

**Affiliations:** 1 Science, Evidence and Analytics Directorate, National Institute for Health and Care Excellence, London, UK; 2 Commercial Medicines Directorate, NHS England and NHS Improvement, Leeds, UK; 3 Department of Development, Science and International Affairs, Zorginstituut Nederland, Diemen, The Netherlands; 4 Syreon Research Institute, Budapest, Hungary; 5 Division of Pharmacoepidemiology and Clinical Pharmacology, Utrecht Institute for Pharmaceutical Sciences, Utrecht University, Utrecht, The Netherlands

**Keywords:** Coronavirus, technology assessment, biomedical, cost effectiveness, pandemics

## Abstract

**Objectives:**

To develop best-practice guidance for health technology assessment (HTA) agencies when appraising diagnostic tests for SARS-CoV-2 and treatments for COVID-19.

**Methods:**

We used a policy sandbox approach to develop best-practice guidance for HTA agencies to approach known challenges associated with assessing tests and treatments for COVID-19. The guidance was developed by a multi-stakeholder workshop of twenty-one participants representing HTA agencies, clinical and patient experts, academia, industry, and a payer, from across Europe and North America. The workshop was supported by extensive background work to identify the key challenges, including: targeted reviews of existing COVID-related methods guidance for assessing interventions and clinical guidelines, engagement with clinical experts, a survey and workshop of HTA agencies, a systematic review of published economic evaluations, and a workshop of health economic modelers.

**Results:**

We suggest HTA agencies should consider using other types of evidence (e.g., real world) where high-quality randomized controlled trials may be lacking and healthcare systems would value timely HTA outputs. A “living” HTA approach may be useful, given the context of an evolving disease, scientific understanding and evidence base, allowing for decisions to be efficiently revisited in response to new information; particularly, if supported by a common “disease model” for COVID-19. Innovative ways of engaging with the public and clinicians, and early engagement with regulators and payers, are recommended.

**Conclusions:**

HTA agencies should consider the elements of this guidance that are most suited to their existing processes to enable them to assess the effectiveness and value of interventions for COVID-19.

## Introduction

Health technology assessment (HTA) has emerged as a discipline that aims to use the best available evidence to inform healthcare systems’ decisions about how to allocate scarce resources to achieve the maximum possible value. The traditional HTA processes of thorough evidence review and careful deliberation were not well suited to informing the rapid healthcare decision making by governments to curb the impact of COVID-19 during 2020 and 2021. Other scientific disciplines provided crucial, timely contributions, including health-related (like epidemiology, public health, immunology, and virology) and others (like behavioral science, logistical modeling, and constrained-optimization modeling). The limited role for HTA may be explained by some as an rational application of the “rule of rescue” (1), while in some countries, there are inherent sensitivities around using economic evaluation to guide health-related resource allocation (2). Additionally, HTA professionals have never faced a situation like COVID-19, and some may have been uncertain about how applicable their standard assessment methods and processes would be during a pandemic (3). Many of these reasons are understandable. However, the absence of value assessments may have in part led to the observed inefficient allocation of resources on some COVID-19 interventions that were later proven to be of limited, if any, value to population health (4;5).

We argue that the core rationale for using HTA to guide decisions relating to the adoption of health technologies is immediately relevant, and even necessary, in the ongoing pandemic context (6), particularly as healthcare systems and budgets return to normal. However, we hypothesized that COVID-19 would expose areas of existing HTA practice that could be improved, and posit that traditional processes may need some modifications to improve agencies’ flexibility and responsiveness. Modifications are proposed as part of best-practice guidance recommendations.

The guidance is intended to support HTA bodies to undertake appraisals of therapeutics for COVID-19 and diagnostics for SARS-CoV-2. Medicines to treat COVID-19 are particularly likely to fall under the remit of most HTA agencies (7), and diagnostic tests featured prominently in decision making during the first years of the pandemic. Other types of intervention, such as vaccines and public health measures, are less likely to be assessed by HTA agencies going forward, and may have important nuances that would distract from the recommendations that are most relevant to most agencies. We propose that HTA agencies give due consideration to this guidance in their assessments, and maintain a robust approach while allowing appropriate pragmatism to adapt to the quickly changing decision context (8). We hope it also helps to enable HTA bodies to prepare a pandemic strategy and operational plan that adopts a living, life-cycle approach, to respond to a future pandemic, epidemic, or other healthcare-related emergency.

The guidance has been developed as part of Next Generation Health Technology Assessment (HTx). HTx is a Horizon 2020 project supported by the European Union lasting for 5 years from January 2019. Its main aim is to create a framework for the next generation of HTA to support patient-centered, societally oriented, real-time decision making on access to and reimbursement for health technologies throughout Europe.

## Background

### COVID-19 poses challenges for existing HTA processes

To understand the HTA challenges posed by COVID-19 and assess the need for modified or novel HTA processes, we used a multifaceted approach. First, to identify the key challenges, we sent a survey to forty-seven HTA agencies, composed of European agencies identified from the European Network for Health Technology Assessment (7) and a similar recent HTx survey (9), and non-European agencies known to have been engaged in COVID-19 assessments. It was completed by twenty-one HTA agencies (sixteen from Europe, three from Australasia, and two from North America), of whom eleven respondents agreed to join a follow-up roundtable workshop. The survey and its findings have been fully reported elsewhere (10). In short, thorough HTA – especially cost-effectiveness analysis – was not a priority for policy makers in the early stage of the pandemic, due to: the need for governments and health systems to act quickly; uncertainty about traditional concepts of “value for money” during a health emergency, and in the context of a rapid expansion of central funds; and the risk of some treatments being subject to a shortage of global supply. HTA representatives perceived key challenges to assessing tests and treatments for COVID-19 as being the lack of high quality, comparative, long-term clinical evidence; nontraditional reporting formats, such as press releases without peer review (11); and heterogeneity in study populations, settings, and outcomes.

Additionally, the COVID-19 context of rapidly evolving scientific understanding and clinical practice meant HTA agencies’ usual “static” decision-making processes could become out of date quickly without a commitment to regular updates. They also faced increased public focus relating to COVID-19 decision making, which created pressure to rapidly approve promising technologies (12) despite uncertain evidence.

To identify particular barriers to assessing the value for money of therapeutics and diagnostics for COVID-19, we also convened a workshop of thirteen health economists and modelers. Participants were identified as economists known by the authors to be engaged in COVID-19 modeling, and were from academia (*n* = 9) or HTA (*n* = 4) in a range of countries (three from England, three from the Netherlands, two from Canada, two from US, one from Hungary, one from Scotland, and one from Ukraine). We also conducted a systematic review of published economic evaluations of COVID-19 therapeutics and diagnostics, to learn from early attempts to assess such technologies; its methods and results have been reported elsewhere (13). From these activities, it was clear that early COVID-19 modeling efforts had lacked the high-quality data needed to inform the model structure and parameter inputs. Scientific understanding and the clinical pathway were developing quickly, but relatively little research has focused on the resource use, quality of life and long-term outcomes required by cost-effectiveness models (14). The outputs of economic evaluations using incomplete or poor-quality evidence are less informative to decision makers. Therefore, many modeling efforts have necessarily been exploratory or highly uncertain, which would result in uncertain HTA deliberations. Even when good data are available, rigorous economic evaluations are generally time consuming, which is not consistent with the need to act fast in response to a health emergency. Additionally, to fully understand the value of effective interventions for an infectious disease pandemic, it may be necessary to capture complex and nontraditional modeling elements, such as disease transmission and system capacity effects.

### HTA agencies would value best-practice guidance for COVID-19

**Figure 1.**
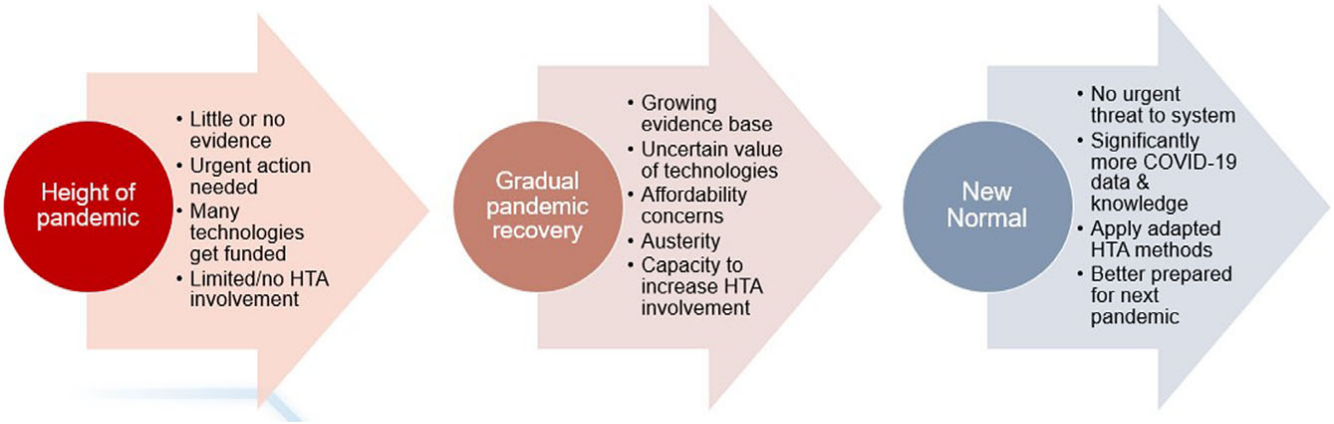
Potential stages of an infectious disease pandemic for HTA agencies.

In many countries, the pandemic context has now shifted away from the urgent situation of 2020 and 2021. Vaccination programs have taken effect and populations have developed a level of immunity to the virus. Healthcare systems are no longer at breaking point, and many novel and repurposed interventions are in development for moderate and severe disease. Management of COVID-19 is returning to the responsibility of traditional, fixed healthcare budgets, as emergency government funding is removed and economies recover (see Figure 1). We conducted a targeted search for existing methods guidance about how to assess technologies for COVID-19 on the web sites of five regulatory bodies (from Australia, Canada, the EU, UK, and US) and forty-two HTA organizations (from twenty-five European countries (*n* = 35) and five non-European countries (*n* = 7)) on 16 March 2021. This identified little methods guidance specifically for COVID-19 technologies; just four documents, from two regulators and one HTA agency, mostly reiterating traditional best practices (15;16). This may be inappropriate, as HTA representatives at our roundtable considered that many of the COVID-19-related challenges are likely to remain for some time, meaning assessments of COVID-19 technologies are unlikely to be straightforward (14;17). Some challenges are not new, such as uncertainty about long-term outcomes, but will continue to exist in a context of wider COVID-related instability. Therefore, best-practice guidance to support HTA agencies would be useful, to: (i) make agencies aware of the myriad issues that they should consider; (ii) provide a framework for consistent assessments, balancing the rigor and pragmatism; and (iii) help prepare HTA processes for potential future pandemics.

## Methods

Following the survey of HTA agencies, roundtable discussion, workshop with health economists and reviews of economic evaluations and existing methods guidance – to identify gaps in HTA processes for the assessment of interventions for COVID-19 – we sought to develop novel guidance to help HTA agencies approach the challenges presented by the pandemic.

We used a “policy sandbox” approach (18) to co-develop the recommendations with multiple HTA stakeholders. Sandboxes provide a way of framing engagement where the objective is to develop new methods, services or processes, by creating a “safe space” to consider the problems and innovate solutions without affecting ongoing work (e.g., this process did not affect the concurrent NICE COVID-19 rapid evidence reviews (19)). For this, we convened a multi-stakeholder group composed of twenty-one participants representing HTA agencies (*n* = 7), clinicians (*n* = 3), academia (*n* = 3), industry (*n* = 3), patient experts (*n* = 2), the Professional Society for Pharmacoeconomics Research (*n* = 2), and a national payer (*n* = 1). Representatives were from countries with various income levels across Europe and North America (4), to make the key principles of the guidance applicable to different economic settings. Stakeholders were first presented with findings from the background work, such as the key challenges for HTA and economic modeling. They were then presented with proposed guidance to address those challenges, which had been drafted by the HTx project team as a “straw man” to encourage discussion. Stakeholders critiqued, revised, removed, and added to the recommendations, at a workshop that held on 5 August 2021 and through subsequent engagement by email, thereby co-developing the final best-practice guidance for HTA agencies.

Separately, we conducted a targeted review of known existing clinical guidelines and pathways (19–27), and engaged with clinical experts known to the authors, to identify the most important outcomes associated with COVID-19 and form a clear understanding of the disease and clinical pathway. The eight clinicians were from Australia, the Netherlands, and UK, and covered the following medical specialties: emergency and critical care, general practice, infectious disease, intensive care, and rehabilitation. The purpose of this was to include a schematic alongside the best-practice guidance recommendations, as a starting point for (i) HTA agencies to consider where interventions may be positioned in the clinical pathway, and what outcomes are most important there, and (ii) the development of future COVID-19 decision models.

## Results

### Key recommendations for HTA

The best-practice guidance represents the consensus view of the multi-stakeholder “COVID-19 HTA best-practice development group,” derived from the sandbox process as described above. Its intended primary audience is HTA agencies planning or undertaking assessments of tests for SARS-CoV-2 or treatments for COVID-19. The recommendations provide pragmatic approaches to consider when appraising technologies for COVID-19 as their value for money becomes a more important policy objective. Developers of COVID-19 technologies can also use the guidance to inform their evidence generation and submission dossiers, while it can help inform other stakeholders in their engagement with, and expectations of, HTA processes.

The key recommendations are summarized below, and available in full at: https://www.htx-h2020.eu/publications/ (“Best-practice guidance for the health technology assessment of diagnostics and treatments for COVID-19”) (8).

#### Assessing clinical effectiveness

Randomized controlled trials (RCTs) should remain the gold standard for clinical evidence for technologies for COVID-19. However, during a pandemic, especially in the early stages, high-quality long-term RCT data are likely to be lacking, while evidence from traditionally less robust sources may be generate more quickly (such as real-world evidence (RWE), studies from other settings, studies published as pre-prints). In these circumstances, HTA agencies should be open-minded about incorporating such evidence into their assessments technologies for COVID-19; weighing the benefit to the healthcare system of timely HTA guidance at a time of great need, against the risk that the available evidence is too weak for accurate decision making. To increase their confidence in doing so, agencies should utilize and engage with the many European research initiatives seeking to enhance the availability of RWE and developing methods to analyze it appropriately (such as HTx, European Health Data and Evidence Network, GetReal Institute, and RWE4Decisions). Pragmatically, agencies should also consider using existing “living” systematic reviews to inform their clinical effectiveness assessments (28–30), to save time and resources and avoid duplication of efforts. Similarly, published core outcome sets for COVID-19 could be used to identify the most appropriate clinical outcomes (28;31).

#### Assessing value for money

HTA agencies that use cost–utility analysis should continue to do so when assessing technologies for COVID-19. Other types of economic evaluation, such as cost–consequences analysis, can also be considered, where they are likely to be useful. Both the healthcare (payer) perspective and the societal perspective should be considered relevant to decision making about COVID-19 technologies. A societal perspective is likely to be more important when the pandemic situation is urgent (32) and when funding is provided from a central government, rather than a specific healthcare budget. HTA agencies should continue to use their standard cost-effectiveness thresholds in COVID-19 assessments for now, but should engage in research to identify whether there are different societal preferences during and following a pandemic.

#### Economic modeling

To inform assessments of cost effectiveness across a wide range of COVID-19 technologies, or sequences of technologies, the use of a whole-disease pathway model including both diagnosis and treatment is recommended. The model should ideally be an individual-level simulation to capture the heterogeneous patient population, the complex pathway, and the potential impact of some technologies on transmission or system capacity.

It should accommodate a wide range of options, including allowing it to run an individual-level or cohort-level model, capture disease transmission (e.g., by linking to an epidemiological or infectious disease model), and incorporate system capacity effects. Capacity effects will be more important to consider during surges of infection that lead to increased pressure on healthcare resources beyond their capacity constraints. The model should also include the flexibility to disable any of these options, to facilitate a less complex modeling approach where appropriate. For example, a simple cohort-level model may be acceptable when assessing a straightforward, narrowly defined decision problem, or multiple technologies at the same position in the treatment pathway. Any economic model should capture the long-term COVID-19 outcomes (33;34) and treatment effects.

**Figure 2.**
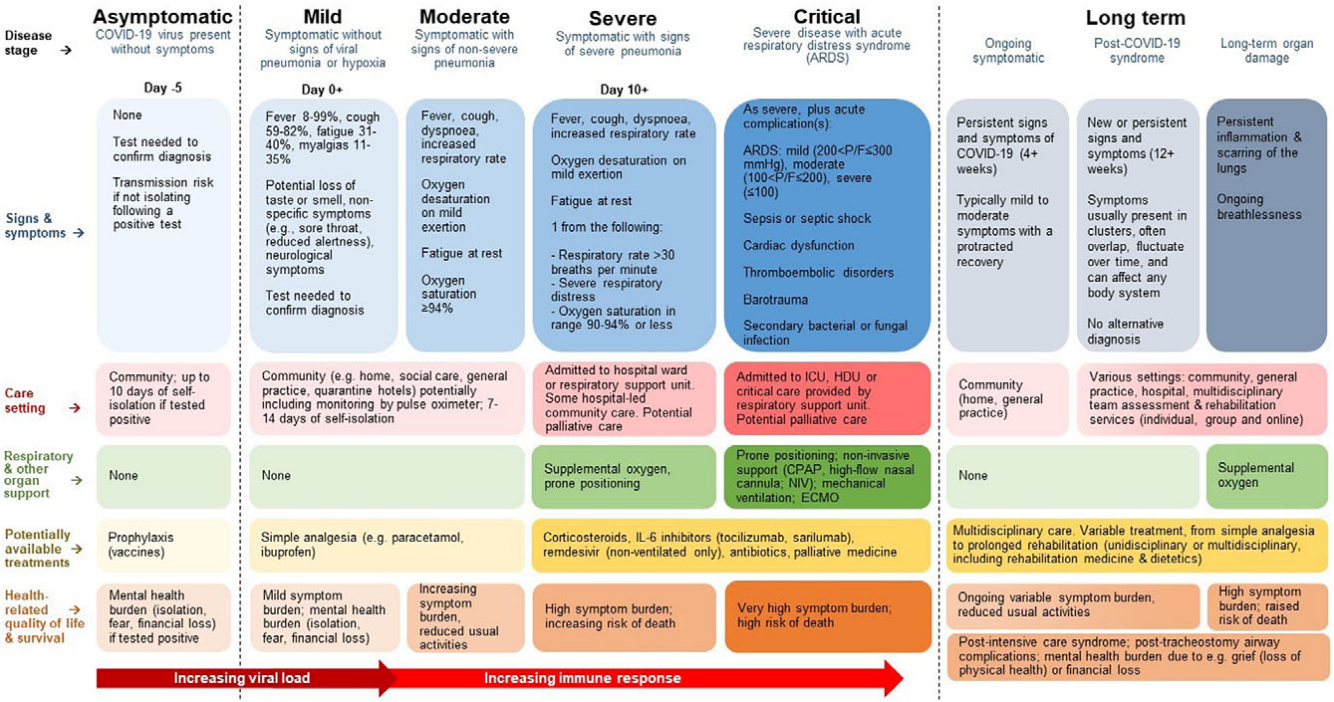
COVID-19 clinical and disease pathway derived from clinical guidelines and expertise.

The model should be frequently updated to reflect the most up-to-date understanding of the disease and changes in the standard of care. A “living,” adaptable whole-disease pathway model could be collaboratively developed by HTA agencies, with input from multiple stakeholders, and made freely available for use and adaptation to support a globally responsive HTA approach to COVID-19 technologies. As a starting point for such efforts, we have developed a summary COVID-19 disease and clinical pathway, derived from clinical guidance and expertise (see Figure 2).

#### Considering uncertainty

Most COVID-19 assessments will have a high level of uncertainty. Agencies should be transparent about the data gaps and assumptions made and should consider the results of extensive sensitivity and threshold analyses. Probabilistic results should still be used and could be accompanied by value of information analysis to inform future research needs. A transparent, pragmatic, “living” HTA approach should be implemented, with a commitment to responsively reviewing decisions as new and better evidence emerges, including potentially reversing previous decisions by reinvesting or disinvesting in technologies.

#### Affordability and procurement

HTA agencies should routinely consider the expected budget impact (35) of COVID-19 technologies, including any required service redesign and system burden, to identify technologies that would be difficult for the healthcare system to implement. This could trigger commercial discussions between the technology developer and healthcare payer. Alternatively, it could trigger the HTA agency to explore subgroup analyses, to identify groups for whom a technology is most cost effective and inform the prioritization of its use. Commissioners and payers may explore novel payment models for COVID-19 technologies. Managed access agreements, including a period of data collection to resolve key uncertainties, may be particularly well supported by a living HTA approach.

#### Considering other elements of value

HTA agencies should consider whether there are relevant benefits that are not adequately captured in the clinical and cost-effectiveness assessments of COVID-19 technologies, such as reduced inequity, reduced fear of infection, and scientific innovation. If it is not possible to include these effects quantitatively (e.g., by capturing them in utility values), then they should be considered using a qualitative and deliberative approach.

#### Stakeholder engagement

The far-reaching effects of the pandemic mean HTA agencies should ensure a broad range of stakeholders can contribute to the assessment of COVID-19 technologies. Input from clinical and patient experts may be particularly informative where there are evidence gaps and uncertainties. A tiered approach to patient and public engagement is recommended, including circumstances to approach citizens’ groups and organizations that represent high-risk groups. HTA agencies could consider using innovative methods to facilitate broad engagement, such as digital and online communication tools.

## Conclusions

The pandemic has undoubtedly created challenges for HTA agencies, and many of these will persist for some time. To address them, we encourage agencies to formally consider this best-practice guidance when assessing technologies for COVID-19, and to implement its recommendations where possible. It has been co-developed with a range of stakeholders, providing a framework to support timely, evidence-based decisions about the value of the many technologies for COVID-19 in the coming months and years, as healthcare systems move away from the emergency pandemic response and once again seek cost-effective, affordable options. In the event of a future infectious disease pandemic, the guidance may also help HTA agencies to contribute more quickly, by planning their assessments and modeling requirements earlier and preparing for more flexible, responsive decision making. We acknowledge that not all elements of the guidance will be applicable for every HTA agency, but individual agencies may still wish to adopt the recommendations that are most suited to their preferred processes and methods.

While this guidance will support the medium-term response of HTA agencies to the pandemic, traditional HTA approaches, like cost-effectiveness analysis, might not be optimal or practical during the initial, emergency response to healthcare crises. Further research, perhaps using a policy sandbox approach, to explore the best ways for HTA agencies to provide an immediate contribution at such times – including for other types of intervention, such as vaccines and public health strategies – would be valuable.

## References

[1] McKie J, Richardson J. The rule of rescue. Soc Sci Med. 2003;56(12):2407–2419. 12742604 10.1016/s0277-9536(02)00244-7

[2] Neumann PJ. Why don’t Americans use cost-effectiveness analysis? Am J Manag Care. 2004;10(5):308–312. 15152700

[3] Padula WV, Reid N, Tierce J. Health technology assessment for COVID-19 treatments and vaccines: Will cost-effectiveness analysis serve our needs? Value Outcomes Spotlight. 2021;7(1):23–25.

[4] Dawoud D, Chalkidou K, Sullivan R, Ruiz FJ, Adler A. USA stockpiling of remdesivir: How should the world respond? J Comp Eff Res. 2020;9(18):1243–1246. 33274643 10.2217/cer-2020-0174 PMC7717394

[5] Campbell J, Whittington M, Rind D, Pearson S. Alternative pricing models for remdesivir and other potential treatments for COVID-19. Updated Report, Institute for Clinical and Economic Review; 2020.

[6] Kaló Z, Németh B, Zemplényi A. Can cost-effectiveness principles be ignored in urgent times? J Comp Eff Res. 2022;11:7–9. 34668766 10.2217/cer-2021-0211 PMC8528146

[7] EUnetHTA. An analysis of HTA and reimbursement procedures in EUnetHTA partner countries. Final report; 2017 (14/01/2017).

[8] Elvidge J, Summerfield A, Knies S, et al. Best-practice guidance for the health technology assessment of diagnostics and treatments for COVID-19. Zenodo; 2021 (27/09/2021).

[9] Hogervorst M, Vreman R, Quik E, Mantel-Teeuwisse A, Goettsch W. Systematic review on how (cost-) effectiveness of combinations of health technologies, individualised/personalised treatments and treatment pathwwys is currently being assessed by HTA bodies. Project deliverable. Next Generation Health Technology Assessment. Report No.: D1.1; 2020 (30/04/2020).

[10] Elvidge J, Dawoud D. Assessing technologies for COVID-19: What are the challenges for health technology assessment agencies? Findings from a survey and roundtable workshop. PharmacoEconomics. 2021;39:1455–1463. 34635993 10.1007/s40273-021-01097-4 PMC8505112

[11] Press Release. Gilead Announces Results From Phase 3 Trial of Investigational Antiviral Remdesivir in Patients With Severe COVID-19. [Press Release]; 2020.

[12] O’Rourke B, Orsini L, Guerino J. COVID-19: Challenges and opportunities for the global health technology assessment community. Value Outcomes Spotlight. 2021;7(3):23–25.

[13] Elvidge J, Summerfield A, Nicholls D, Dawoud D. Diagnostics and treatments of COVID-19: A living systematic review of economic evaluations. Value Health 2022;25(5):773–784. 35181207 10.1016/j.jval.2022.01.001 PMC8847103

[14] Lorgelly PK, Adler A. Impact of a global pandemic on health technology assessment. Appl Health Econ Health Policy. 2020;18(3):339–343. 32377982 10.1007/s40258-020-00590-9 PMC7202921

[15] NICE. Support for developers of medicinal products for COVID-19. 2020. https://www.nice.org.uk/covid-19/support-for-developers-of-medicinal-products-for-covid-19

[16] (FDA) UFaDA. COVID-19: Developing drugs and biological products for treatment of prevention. Guidance for Industry; 2020.

[17] O’Rourke B, Oortwijn W, Schuller T. The new definition of health technology assessment: A milestone in international collaboration. Int J Technol Assess Health Care. 2020;36(3):187–190. 32398176 10.1017/S0266462320000215

[18] Leckenby E, Dawoud D, Bouvy J, Jónsson P. The sandbox approach and its potential for use in health technology assessment: A literature review. Appl Health Econ Health Policy. 2021;19(6):857–869. 34254275 10.1007/s40258-021-00665-1 PMC8545721

[19] NICE. COVID-19 rapid guideline: Managing COVID-19. NICE Guideline. NG191; 2021.

[20] NICE. COVID-19 rapid guideline: Managing the long-term effects of COVID-19. NICE Guideline. NG188; 2021. 33555768

[21] NHS. Clinical guide for the management of emergency department patients during the COVID-19 pandemic; 2021. https://www.nice.org.uk/covid-19/specialty-guides

[22] World Health Organization (WHO). COVID-19 therapeutic trial synopsis; 2020. https://www.who.int/publications/i/item/covid-19-therapeutic-trial-synopsis

[23] World Health Organization (WHO). COVID-19 clinical management: Living guidance; 2021. https://www.who.int/publications/i/item/WHO-2019-nCoV-clinical-2021-2

[24] Messer B, Allen M, Antoine-Pitterson P, et al. Respiratory support units - guidance on development and implementation. Br Thoracic Soc. 2021;12:1–23.

[25] Cevik M, Kuppalli K, Kindrachuk J, Peiris M. Virology, transmission, and pathogenesis of SARS-CoV-2. BMJ. 2020;371:m3862. 33097561 10.1136/bmj.m3862

[26] Stam H, Stucki G, Bickenbach J. Covid-19 and post intensive care syndrome: A call for action. J Rehabil Med. 2020;52(4):jrm00044. 32286675 10.2340/16501977-2677

[27] National Institutes of Health – COVID-19 Treatment Guidelines Panel. Coronavirus disease 2019 (COVID-19) treatment guidelines; 2021. https://www.covid19treatmentguidelines.nih.gov/34003615

[28] Boutron I, Chaimani A, Meerpohl JJ, et al. The COVID-NMA project: Building an evidence ecosystem for the COVID-19 pandemic. Ann Intern Med. 2020;173(12):1015–1017. 32931326 10.7326/M20-5261 PMC7518109

[29] EUnetHTA. COVID-19 treatments - Rolling Collaborative Reviews; 2021. https://www.eunethta.eu/covid-19-treatment/

[30] Siemieniuk RA, Bartoszko JJ, Ge L, et al. Drug treatments for covid-19: Living systematic review and network meta-analysis. BMJ (Clinical research ed). 2020;370:m2980. 32732190 10.1136/bmj.m2980 PMC7390912

[31] COMET Initiative. Core outcome set developers’ response to COVID-19; 2021. https://www.comet-initiative.org/studies/details/1538

[32] Schnitzler L, Janssen LMM, Evers SMAA, et al. The broader societal impacts of COVID-19 and the growing importance of capturing these in health economic analyses. Int J Technol Assess Health Care. 2021;37:e43. 33686927 10.1017/S0266462321000155

[33] NHS. Long-term effects of coronavirus (long COVID); 2021. https://www.nhs.uk/conditions/covid-19/long-term-effects-of-covid-19-long-covid/

[34] World Health Organization (WHO). Clinical long-term effects of COVID-19; 2021. https://www.who.int/publications/m/item/update-54-clinical-long-term-effects-of-covid-19

[35] Sullivan SD, Mauskopf JA, Augustovski F, et al. Budget impact analysis-principles of good practice: Report of the ISPOR 2012 budget impact analysis good practice II task force. Value Health. 2014;17(1):5–14. 24438712 10.1016/j.jval.2013.08.2291

